# Cross-Sectional Survey on the Relationship Between Occupational Stress, Hormone Levels, and the Sleep Quality of Oilfield Workers in Xinjiang, China

**DOI:** 10.3390/ijerph16183316

**Published:** 2019-09-09

**Authors:** Xue Li, Xiaoyan Gao, Jiwen Liu

**Affiliations:** Department of Public Health, Xinjiang Medical University, Urumqi 830011, China (X.L.) (X.G.)

**Keywords:** occupational stress, sleep quality, oilfield workers, glucocorticoid

## Abstract

The impact of psychosocial factors on health has received increased attention. This study employed a multi-stage hierarchical cluster sampling method and a cross-sectional survey was conducted from March to August 2017. By studying 2116 oilfield workers based in Karamay, Xinjiang, the relationship between occupational stress, blood hormone levels, and sleep was analyzed. Occupational stress was measured using the internationally accepted Occupational Stress Inventory Revised Edition (OSI-R) questionnaire and sleep disorders were measured using the Pittsburgh Sleep Quality Index (PSQI) questionnaire. The study found that the sleep quality of respondents was not high and the incidence of sleep disorders was 36.67%. The higher the level of occupational stress, the higher the incidence of sleep disorders. Irregular shifts can affect sleep quality and individuals with high-level professional titles experience a higher incidence of sleep disorders than those with low-level titles. The total score of the PSQI was different among the low, medium, and high stress groups. The higher the level of stress, the higher the scores of subjective sleep quality, sleep disorder, and daytime dysfunction. The scores of the PSQI, subjective sleep quality, sleep time, sleep disturbance, and daytime dysfunction in the high-stress group were higher than those in the low stress group. A case-control study found that the concentration of glucocorticoids in the sleep disorder positive group was lower than that in the sleep disorder negative group. The results of the regression analysis showed that glucocorticoid is a protective factor for sleep disorders (OR = 0.989, 95% CI: 0.983–0.995), suggesting that the higher the level of glycosaminoglycan, the less likely the subject is to have sleep disorders. For example, in the case of high occupational stress, the interaction between low and moderate occupational stress levels and glucocorticoids is a protective factor for sleep disorders.

## 1. Introduction

At present, social psychological factors have become an important factor affecting the physical and mental health and work ability of occupational groups. The social psychological factors that are observed in the workplace (e.g., occupational stress, burnout, anxiety, depression, etc.) are a hot issue in current international occupational health discipline research [1,2,3]. The World Health Organization stated that occupational stress has become a worldwide epidemic and is a common concern among many disciplines [4]. Occupational stress is also termed work stress, and refers to the imbalance between the objective needs of workers and the ability of individuals to adapt to work, resulting in physical and psychological stress [5]. Studies have shown that tension is associated with a range of sub-health performances, which can lead to a variety of diseases, such as cardiovascular disease, digestive diseases, insomnia, depression, anxiety, suicide, and overworking. Furthermore, the stress mechanism is closely related to the occurrence of more serious fatal diseases [6]. Mosadeghrad and Habibi suggested that an increasing level of stress seriously affects the physical and mental health of workers, reducing their ability and diminishing their quality of life [7,8]. In recent years, the results of research carried out at home and abroad have shown that long-term stress can increase the number of the body’s self-regulatory responses during the night, thus, reducing sleep quality [9,10]. When excessive job stress exceeds the body’s own ability to adjust, an imbalance in the body’s functioning results, which affects the sleep of the occupational population and results in a decline in sleep quality, as well as sleep problems such as insomnia and lethargy [11,12]. Studies have shown that sleep problems are an early symptom of many physical and mental illnesses [13,14]. For the professional population, many occupational stress-related factors have a significant negative impact on sleep, such as high job requirements, low work control [15], and high-pay-low-return situations [16]. Occupational stress is a major occupational risk factor for increasing the risk of sleep problems [17,18]. Some studies carried out abroad suggested that based on occupational health theory, work stress and poor social working conditions are considered to be important determinants of poor sleep quality [19,20,21]. Nixon et al. [22] conducted a meta-analysis of the effects of occupational stress on physical health and found that factors such as excessive workload, conflicting roles, vague roles, and interpersonal conflicts were closely related to sleep quality. If sleep problems occur in the occupational population, the number of people taking sick leave increases, while work efficiency decreases and work-related accidents increase [23], which has a huge impact on the economic benefits of enterprises.

At present, research on sleep problems has been extended into the domains of epidemiology, neurobiological mechanisms, and etiology [24,25,26,27]. Melatonin (MLT) is a neuroregulatory hormone secreted by the pineal gland. MTL secretion and sleep control are both autonomous rhythm processes. MTL plays a decisive role in the photoperiod phenomenon and can act on photoperiodic organisms. In turn, it affects the physiological activities of the organism. Studies have shown that [28] by acting on brain-related physiological activities, MTL can assist the brain by encouraging a deeper sleep, reducing the frequency of awakening during sleep, enabling the brain to get enough rest, significantly improving physiological discomfort, and also adjusting the drowsiness caused by jet lag. Moreover, an age-related decline in melatonin can lead to sleep disorders and metabolic circadian rhythm disorders [29]. Glucocorticoids, referred to as “corticosteroids”, are hormones synthesized and secreted by adrenal bundles. The secretion of glucocorticoids also has circadian rhythm. It is primarily secreted between 8–10 am, then gradually decreases, and the secretion is at its lowest level at 12 pm. The study found that sleep activity plays an important role in regulating the hypothalamic-pituitary-adrenal (HPA) axis [30]. Therefore, insomnia is closely associated with HPA axis hormones. As an HPA axis hormone, abnormal changes in glucocorticoids and GH levels may result in insomnia. During sleep, serum glucocorticoid levels were significantly reduced and GH levels were significantly elevated [31]. Cortisol is also an important stress hormone secreted by the adrenal gland. It is a neurohormone and its secretion has the lowest circadian rhythm at night and the highest level in the morning. Related studies suggested that cortisol hyperfunction can inhibit sleep in the case of a stress response [32,33] and sleep deprivation can activate the pituitary-hypothalamic-adrenal axis, leading to elevated cortisol levels [34]. Alderling et al. [35] observed different cortisol concentrations during the period of awakening, at half an hour post-awakening, at lunch time, and before going to bed. At half an hour post-awakening, the concentration of cortisol reached its peak, while the cortisol concentration was at its lowest before going to bed. Furthermore, the study found that during the post-awakening period, the concentration of cortisol in women with sleep disorders was significantly lower than that in the healthy group.

At present, most of China’s oilfield exploitation areas are located in the Gobi Desert where the natural environment is poor and oilfield workers are far from the city and their families. They suffer from a long-term lack of face-to-face emotional communication with relatives and friends and they also have to endure the harm caused by the inherent nature of work shifts and its adverse impact on sleep. A poor working environment and irregular working hours have a negative impact on the physical and mental health of oilfield workers and increase the risk of disease [36,37]. While some studies have shown that occupational stress has an impact on sleep quality, few studies have examined the relationship between occupational stress, hormone levels, and sleep. Therefore, this study investigated occupational stress, hormone levels, and the sleep quality of oilfield workers based in Karamay City, Xinjiang, and analyzed the effects of tension and hormone levels on sleep.

## 2. Materials and Methods

### 2.1. Subjects

The study was carried out in the Department of Occupational Diseases and Physical Therapy at the Central Hospital of Karamay, Xinjiang. After having communicated with the hospital at the early stage of the study, the content of the questionnaire was used as part of the occupational health examination of petroleum workers. The study was carried out from March to October 2017. The research object of this study included all employees from the China National Petroleum Corporation (CNPC) at the Xinjiang Petroleum Administration Bureau in Karamay City, Xinjiang. The Administration has 25 subordinate units and approximately 150,000 employees and covers all work related to the oil industry. A three-stage stratified sampling method was employed. According to the China National Petroleum Corporation, four operating areas, four production plants and six exploration and development companies were selected. Depending on the size of the company, a large company (>400 workers) and a small company (<400 workers) were randomly selected from the chosen business areas; one large company (>1000 workers) and one company selected from among the small production plant companies (<1000 workers); one large company (>200 workers) and two small companies (<200 workers) from the exploration and development company. Before the medical examination, the hospital obtained a list of personnel and randomly selected the respondents from the list. In respect to the business area, 500 and 200 people were randomly selected from large companies and small companies, respectively. For the production plants, 800 and 500 people were randomly selected from large and small companies, respectively. For exploration and development companies, 130 people were randomly selected from large companies and 85 people were selected from each of the two small companies. Subjects included those who satisfied the following criteria: Randomly selected oil workers aged 18–60 years old and who were employed for more than one year. A total of 2300 questionnaires were distributed and 2116 valid questionnaires were finally retrieved. The effective recovery rate was 92%. The research protocol was approved by the Ethics Committee of Xinjiang Medical University. Before commencement of the investigation, all respondents voluntarily provided their written informed consent. In this study, a total of 776 people with positive sleep disorders were screened, accounting for 36.67% of the total. The target of the general index measurement was 10–20% of the randomly selected questionnaire respondents, which represented the experimental research object. Therefore, in this study, 20% of participants who were positive for sleep disorders were randomly selected as test-subjects for sleep-related hormone levels and a 1:1 match was used to select a case-control study with a positive sleep disorder participant who was negative for sleep disorders, within an age range of ±1 year, who was the same sex, and from the same ethnic group. Finally, there were 154 experimental participants and 154 control samples.

### 2.2. Measures

#### 2.2.1. Occupational Stress

The Occupational Stress Inventory Revised Edition (OSI-R) was designed by Osipow in 1998 [38] and the scale was introduced to China in 1999. In order to address any misunderstanding caused by economic, cultural or customary differences, and to improve the value of the scale’s application in China, it was translated and revised several times under the guidance of relevant experts. In addition, some of the items were randomly selected for back-translation and modified again. The content expressed in the original text was accurately understood. However, while considering China’s cultural background, economic development level, and people’s health awareness, 30 items and questioning methods were selected from among the 140 items in the OSI-R and were revised. The reliability and validity of the scale were verified, and it can be regarded as a professional tool for occupational stress research in China [39]. The OSI-R scale consists of three subscales, as follows: The Occupational Role Questionnaire (ORQ), which includes six sub-items consisting in role overload (RO), role insufficiency (RI), role ambiguity (RA), role boundary (RB), responsibility (R), physical environment (PE), and each sub-item includes ten entries; the Personal Strain Questionnaire (PSQ) consists of four sub-items, which include vocational strain (VS), psychological strain (PSY), interpersonal strain (IS), and physical strain (PHS), and each sub-item includes ten entries; the Personal Resources Questionnaire (PRQ) consists of four sub-items, including recreation (RE), self-care (SC), social support (SS), and rational coping (RC); each sub-item includes ten entries, producing a total of 140 items. Higher ORQ scores indicate more demanding tasks, higher PSQ scores indicate higher levels of stress, and higher PRQ scores indicate a stronger ability to respond to occupational stress. By grouping the subjects’ occupational stress according to the PRQ score [40] and the occupational stress norm [41], based on the formula T = 50 + 10 ($Χ-\bar{Χ}$)/s (i.e., where Χ represents each sub-item, $\bar{Χ}$ is the norm average, and s is the norm standard deviation), the original score of the subscale is converted into the T value. A T value ≥70 is classified as high occupational stress; 60 to 69 points indicate moderate occupational stress; and ≤59 points indicates low occupational stress.

#### 2.2.2. Sleep Quality

Using the Pittsburgh Sleep Quality Index (PSQI), which was developed by Dr. Buyee in 1989 [42], the table has good reliability and validity. According to the Chinese version of the reliability and validity study as well as the study carried out by Tsai et al. [43], the Chinese version of the PSQI (i.e., the CPSQI) has shown good internal consistency and reliability (Cronbach’s α = 0.82–0.83) and the retest reliability of 14–21 d was 0.85 (all subjects) and 0.779 (primary insomnia). The table consists of 19 self-assessments and five other evaluation entries (the five other evaluation entries do not feature in the PSQI total score). The scoring items included subjective sleep quality, time of falling asleep, sleeping time, sleep efficiency, sleep disorders, use of hypnotic drugs, and daytime dysfunction [44,45]. Each item is scored on a scale ranging from 0 to 3. The accumulated scores of each component are PSQI total scores, and the total score ranges from 0 to 21. The higher the score, the worse the sleep quality. According to international standards, a total PSQI score of ≥5 indicates a sleep disorder [46].

#### 2.2.3. Collection of Blood Samples

Participants provided their informed consent, fasted, and were advised to avoid drinking water. Using a 4 mL vacuum blood collection tube, blood samples were collected by medical staff at the medical center from 9 am to 11 am in the morning. Serum and plasma were separated by centrifugation at 3500 rpm/min for 7 min. Samples were stored in a −80 °C refrigerator and prevented from repeated dissolution.

### 2.3. Quality Control

#### 2.3.1. Quality Control of On-Site Epidemiological Investigation

The research team and the cooperation unit established long-term and stable cooperative relationships. During the early stage of the investigation, the researchers communicated with the cooperation unit, formulated a sound investigation process and schedule, and conducted unified training for the investigation of personnel, so that the investigation of personnel could clarify the purpose and significance of the investigation, and satisfy the standardized format and requirements of the questionnaire. Before the survey, the questionnaires were numbered consistently and distributed to the respondents. Before completing the survey, the researchers explained the contents of the questionnaire, as well as what was required of participants to complete the survey. The questionnaires were retrieved in a centralized manner and collected and reviewed immediately. A questionnaire with content below 80% was considered an invalid questionnaire.

#### 2.3.2. Laboratory Quality Control

When testing the laboratory indicators, the researchers ensured that the instrument had no technical failure and it was only used after having verified that it operated effectively. The laboratory’s operating rules were strictly adhered to, disposable laboratory equipment was used, and the researchers avoided contamination of samples and reagents. The experimental process strictly followed laboratory quality control standards and the determination of relevant hormone levels was carried out strictly in accordance with the kit’s instructions. Prior to the experiment, the disposable tip was autoclaved, and it was necessary to preheat the instrument before use. Before the formal experiment was conducted, several samples were used for the pre-experiment, and the experimental results were observed in order to identify and resolve problems promptly to ensure that the formal experiments ran smoothly and were as accurate as possible.

### 2.4. Statistical Analysis

SPSS for Windows v.17.0 software (SPSS Inc., Chicago, IL, USA) was used for data processing and statistical analysis. The measurement data are statistically described by $\bar{X}\pm S$. Two independent samples *t*-tests were carried out to compare the two groups, and a multi-group mean comparison was performed using a one-way ANOVA. If there is a difference in the multi-group mean, the SNK-q and LSD tests can be used for pairwise comparisons. A chi-square test was conducted to compare the rate. The significance level was *α* = 0.05.

## 3. Results

### 3.1. The Incidence of Sleep Disorders in Oilfield Workers with Different Population Characteristics

The incidence of sleep disorders was different for fixed day shifts (31.35%), regular shifts (38.90%), and irregular shifts (45.96%) (*P* < 0.001): Professional titles had an impact on the incidence of sleep disorders (*P* < 0.05). No statistical difference was found in the incidence of sleep disorders among oilfield workers with other demographic characteristics (*P* > 0.05) (Table 1).

### 3.2. Incidence of Sleep Disorders According to Different Occupational Stress Levels

The lowest incidence of sleep disorders was found in the low stress group (26.97%) and the highest incidence of sleep disorders was observed in the high stress group (38.88%). The trend of the chi-square test showed that the prevalence of sleep disorders in the low, medium, and high occupational stress groups was statistically significant (*P* = 0.021) (Table 2).

### 3.3. The Relationship Between Different Occupational Stress Levels and PSQI Sub-Items

The PSQI total score was different between the low, medium, and high stress groups. The higher the level of stress, the higher the scores of subjective sleep quality, sleep disorders, and daytime dysfunction (*P* < 0.001). Differences were found between the low and high stress groups with respect to the PSQI total score, subjective sleep quality, time of fall asleep, sleep disorders, daytime dysfunction (*P* < 0.05) (Table 3).

### 3.4. Relationship Between Blood Hormone Levels and Sleep Disorders

#### 3.4.1. Balance Test

There was no significant difference in the age, sex, and ethnicity of participants in the positive and negative sleep disorders group (*P* < 0.05). Those data suggested that the positive sleep disorder group is comparable to the negative sleep disorder group (Table 4).

#### 3.4.2. Comparison of Hormone Levels in Different Sleep Conditions

Glucocorticoid concentration in the sleep disorder positive group was lower than the negative group, and there was a statistically significant difference (*P* < 0.05). Cortisol and melatonin concentrations were lower in the positive group than in the negative group, and the difference was not statistically significant (*P* > 0.05) (Table 5).

### 3.5. Relationship Between Occupational Stress, Hormone Levels, and Sleep Disorders

The relationship between occupational stress, hormone levels, and sleep disorders was further analyzed, and “with or without sleep disorders” was used as a dependent variable (0 = negative sleep disorder, 1 = sleep disorder positive). Low, medium, and high occupational stress levels and hormone levels were used as independent variables for multivariate logistic regression analysis. The results showed that using low occupational stress group as a reference, moderate and high levels of occupational stress are risk factors for sleep disorders (OR = 3.549, 95% CI.: 1.617–7.788; OR = 8.684, 95% CI.: 3.872–19.473); Glucocorticoids are protective factors for sleep disorders (OR = 0.989, 95% CI.: 0.983–0.995). It is suggested that the higher the glucocorticoid level, the less likely the subject is to suffer from sleep disorders (Table 6).

### 3.6. Effects of the Interaction of Occupational Stress and Hormone Levels on Sleep

Taking the high occupational stress group as a reference, a logistic regression analysis of occupational stress, glucocorticoid interactions, and sleep disorders was conducted. The results showed that the interaction between occupational stress and glucocorticoid levels has an impact on sleep disorders. Taking the high occupational stress group as a reference, the interaction between low and medium occupational stress and glucocorticoids is a protective factor for sleep disorders (Table 7).

## 4. Discussion

Sleep is an essential physiological activity that maintains the normal functioning of the body. With regular and good sleep, the body is relieved of fatigue, and mental and physical strength can be restored to ensure that the body remains in good condition and that health is maintained. At present, the prevalence of sleep problems around the world is very high. Sleep problems have gradually become a health concern that cannot be ignored and have attracted the attention of scholars from various disciplines [47,48,49].

This study found that different shifts affect sleep, and irregular shifts increase the proportion of people with sleep problems. The human body relieves fatigue through sleep, and frequent shifts prevent the human body from getting enough rest; thus, disturbing the biological rhythm of the human body and causing a decline in sleep quality. Pallesen [50] and others believed that frequent shifts and night shifts leave employees with long-term inversion and overload of the clock, which affects their sleep quality, daytime functioning, and quality of life. Workers with high-level professional titles show higher rates of sleep disorders than those with low-level professional titles. This may be because people with high-level professional titles are often in a leadership position and take on more responsibility and pressure. In addition, most of the supervisors are still in working condition after work, and they cannot stop working immediately and have complete rest, which affects sleep. Most low-level professional oil workers follow the supervisor’s arrangement. Although they expend more physical energy during work, they can rest their body and brain after work, so the quality of sleep may be higher than that of their supervisors. Studies have found that age and gender can significantly affect sleep quality [51,52] and such findings differ from the results of the current study. This study found that the incidence of sleep disorders was 36.67% after a survey of sleep quality, which was carried out with 2116 oil workers. As stress increases, the incidence of sleep disorders increases. This study found that as occupational stress increased, the scores of the PSQI total score, subjective sleep quality, sleep disorder, and daytime dysfunction increased. At the same time, the low-level occupational stress group was used as a reference, and the middle and high-level occupational stress groups were compared. The analysis revealed that the four scores of the middle and high occupational stress groups were higher than the low occupational stress group, which was consistent with the findings of Li Xue et al. [53]. As the frequency of individual night shifts increases and the pace of life becomes more irregular, anxiety can easily result in the long run, which only exacerbates bodily fatigue and stress levels.

By investigating offshore oil workers, some scholars have found that due to the special nature of the work environment, the operator has less communication with the outside world, and the long-term lack of emotional communication with relatives and friends can easily lead to nervous reactions [54]. The increase in stress levels affects the working ability of workers and the quality of sleep [55]. The study found that occupational stress is a risk factor for insomnia and has a direct relationship with sleep problems. The higher the level of occupational stress, the more likely it is that an individual will experience sleep problems, such as insomnia [9,12]; sleep problems are a potential risk factor for the induction of various diseases, such as cardiovascular and cerebrovascular diseases, neuropsychiatric diseases, etc. [56].

In the case-control study, the concentrations of cortisol and melatonin were lower in the positive sleep disorder group than in the negative sleep disorder group but the difference was not statistically significant. The study was conducted between 9 am and 11 am in the morning and the subjects were subjected to blood collection under fasting conditions. However, due to the special nature of the work of the oil workers, some subjects directly underwent a physical examination after having finished their night work and their bodies did not get enough rest at night, which may have had an impact on hormone secretion. Therefore, the relationship between cortisol/melatonin levels and sleep condition needs further study. Serkan Het et al. [57] found that cortisol and salivary α-amylase (SAA) analysis of 232 subjects showed that the Trier Social Stress Test (TSST) group showed higher levels of cortisol and SAA compared to the Placebo-TSST (P-TSST) group. The Trier test showed that cortisol levels increased in the face of acute stress but when confronted with chronic stress, workers began to adapt to stress and from a biological perspective, this adaptation involves a successive reduction in the cortisol response. This line of reasoning draws on findings from experimental stress provocation research [58] showing habituation effects on cortisol secretion. Chronic stress leads to the occurrence of sleep disorders and emotional exhaustion. Studies have shown that the cortisol concentration of people with sleep disorders post-awakening is lower than that of normal people [35]. A large number of studies around the world have shown that melatonin has many physiological functions, such as promoting sleep, regulating jet lag, anti-aging, regulating immunity, and anti-tumor [59,60], and can be used to treat sleep disorders in children without any significant side effects [61]. The results of this study showed that the level of glucocorticoids in the positive sleep disorder group was lower than that in the negative sleep disorder group. It is suggested that glucocorticoids are associated with the occurrence of sleep disorders, which is consistent with domestic scholars [62,63]. The logistic regression analysis suggested that glucocorticoids are protective factors for sleep disorders, elevated levels of glucocorticoids and reduced the risk of sleep disorders. This study used a high occupational stress group as a reference to analyze the effect of occupational stress and glucocorticoid interaction on sleep. The results showed that the lower the level of occupational stress, the less likely it is to have a sleep disorder. Studies have shown that glucocorticoid combined with montelukast sodium in children with mild obstructive sleep apnea syndrome can significantly improve the quality of sleep [62]. Intranasal glucocorticoids are the first choice for children with mild obstructive sleep apnea and the treatment effect is significant [63].

## 5. Conclusions

Although research has shown that occupational stress can affect sleep, occupational stress does not fully explain the occurrence of sleep disorders. Therefore, this study explored the relationship between the occurrence of sleep disorders and stress and hormone levels, and the current research results suggest that the interaction between occupational stress and hormone levels is related to the occurrence of sleep disorders. However, because this study is a cross-sectional study, it is impossible to make causal inferences. At the same time, due to workload constraints, 1:1 matching of all sleep disorders was not carried out for the case-control study and only 20% of the subjects were selected. In addition, the research objects should be strictly clarified to further improve the scientific and rational results. For future research, we will consider the effects of controlling adverse factors and use the contour level study to verify the results of the study while clarifying its causal relationship. This study found that job titles and shifts affect the incidence of sleep disorders. As the stress level increases, the total score of the PSQI and the scores of each sub-item increase and the incidence of sleep disorders increases. This finding suggests that there is a close relationship between occupational stress and sleep quality. Therefore, to improve the quality of sleep of oil workers and to promote the physical and mental health of occupational groups, we can start by alleviating tension, improving the working environment of oil workers, optimizing the management of labor organizations, and paying attention to observing workers’ emotional states. We can also improve the health awareness of oil workers and encourage workers to develop a sense of self-care. At the same time, through psychological counseling, physical exercise can be encouraged, the tension of workers can be alleviated, and the health of workers can be comprehensively improved. By comparing hormone levels in different sleep conditions, it was found that glucocorticoid levels have an effect on sleep. The correlation analysis between occupational stress, glucocorticoid levels, and sleep disorders showed that the interaction between occupational stress and glucocorticoids had an effect on the development of sleep disorders. Therefore, an objective assessment of the tension of oilfield workers and the effective detection of glucocorticoid levels in serum can be used as a predictor of sleep disorders.

## Figures and Tables

**Table 1:** Comparison of the incidence of sleep disorders according to different population characteristics (%).

Variables		Number	Sleep Disorders (%)	$\chi^{2}$	*P*
Sex	Male	1020	357 (35.00%)	2.373	0.123
	Female	1096	419 (38.23%)		
Age group, years	≤30	385	131 (34.03%)	3.242	0.198
	30–45	1122	431 (38.41%)		
	>45	609	214 (35.14%)		
Ethnicity	Han	1560	566 (36.28%)	0.391	0.532
	Minority	556	210 (37.77%)		
Working years	≤15	765	269 (35.16%)	1.176	0.278
	>15	1351	507 (37.53%)		
Type of work	Extract oil	253	91 (35.97%)	4.330	0.115
	Oil transportation	737	292 (39.62%)		
	Stoker hot note work	1126	393 (34.90%)		
Shift	Fixed day shift	775	243 (31.35%)	17.937	0.000
	Regular shift	1180	459 (38.90%)		
	Irregular shift	161	74 (45.96%)		
Professional titles	Primary and secondary	1208	411 (34.02%)	8.511	0.004
	Vice-senior and Senior	908	365 (40.20%)		
Educational level	Associate’s degree or below	786	300 (38.17%)	1.203	0.273
	Bachelor’s degree or higher	1330	476 (35.79%)		
Marital status	Single	275	92 (33.45%)	1.410	0.235
	Married	1841	684 (37.15%)		
Monthly income	≤3500	733	262 (35.74%)	0.417	0.518
	>3500	1383	514 (37.17%)		
Smoking	Yes	722	283 (39.20%)	3.006	0.083
	No	1394	493 (35.37%)		
Drinking	Yes	1112	424 (38.13%)	2.141	0.143
	No	1004	352 (35.06%)		

**Table 2:** Incidence of sleep disorders between groups with different occupational stress levels (%).

Grouping	Number	Sleep Disorders (n, %)	$\chi^{2}$	*P*
Low stress group	152	41 (26.97)	7.747	0.021
Moderate stress group	1213	443 (36.52)		
High stress group	751	292 (38.88)		
Total	2116	776 (36.67)		

**Table 3:** Comparison of PSQI between different stress levels of workers in Xinjiang oilfield. ($\bar{X}\pm S$).

Variables	Low Stress Group (n = 152)	Moderate Stress Group (n = 1213)	High Stress Group (n = 751)	*t*	*P*
PSQI total score	5.44 ± 3.53	6.40 ± 3.73	6.70 ± 4.19 *	11.037	0.004
Subjective sleep quality	0.95 ± 0.87	1.24 ± 0.87	1.24 ± 0.93 *	14.911	0.001
Time of fall asleep	1.18 ± 0.89	1.35 ± 0.96	1.37 ± 0.98 *	4.655	0.098
Sleeping time	1.05 ± 0.94	1.07 ± 0.93	1.16 ± 0.96	2.502	0.082
Sleep efficiency	0.24 ± 0.61	0.20 ± 0.49	0.26 ± 0.58	3.921	0.141
Sleep disorders	0.89 ± 0.69	1.12 ± 0.72	1.13 ± 0.72 *	7.315	0.001
Hypnotic drugs	0.23 ± 0.58	0.26 ± 0.63	0.31 ± 0.70	2.442	0.295
Daytime dysfunction	0.90 ± 0.82	1.16 ± 0.91	1.23 ± 0.98 *	14.729	0.001

Note: * Compared to the low stress group (*P* < 0.05).

**Table 4:** Balance test between positive sleep disorder groups and negative sleep disorder groups.

Variables		N	Positive Sleep Disorder (n/%)	Negative Sleep Disorder (n/%)	$\chi^{2}$	*P*
Age group, years	≤30	82	40 (25.97)	42 (27.27)	0.332	0.847
	30–45	139	72 (46.75)	67 (43.51)		
	>45	87	42 (27.27)	45 (29.22)		
Sex	Male	153	81 (52.60)	72 (46.75)	1.052	0.305
	Female	155	73 (47.40)	82 (53.25)		
Ethnicity	Han	211	112 (72.73)	99 (64.29)	2.543	0.111
	Minority	97	42 (27.27)	55 (35.71)		

**Table 5:** Comparison of hormone levels between positive sleep disorder groups and negative sleep disorder groups ($\bar{Χ}\pm S$, mmol/L).

Variables	Cortisol	Melatonin	Glucocorticoid
Positive sleep disorder	508.95 ± 23.769	514.26 ± 91.569	35.25 ± 39.318
Negative sleep disorder	511.36 ± 17.709	531.96 ± 100.944	55.57 ± 43.594
*F*	1.014	2.599	18.424
*P*	0.315	0.108	<0.001

**Table 6:** Logistic regression analysis of occupational stress, hormone levels, and sleep disorders.

Variables	β	$\chi^{2}$	*P*	OR	95%CI.
Constant	−2.650	0.700	0.403	0.071	–
Low stress group	–	29.741	<0.01	–	–
Moderate stress group	1.267	9.977	0.002	3.549	1.617–7.788
High stress group	2.161	27.517	<0.01	8.684	3.872–19.473
Cortisol	0.006	3.793	0.314	1.006	0.994–1.018
Melatonin	−0.003	12.780	0.051	0.997	0.995–1.000
Glucocorticoid	−0.011	0.003	<0.01	0.989	0.983–0.995

**Table 7:** Interaction between occupational stress, glucocorticoid levels, and sleep disorders.

Variables	β	$\chi^{2}$	*P*	OR	95%CI.
Glucocorticoid * High stress group	−	20.710	<0.01	−	−
Glucocorticoid * Low stress group	−0.033	16.251	<0.01	0.968	0.953–0.983
Glucocorticoid * Moderate stress group	−0.008	7.061	0.008	0.992	0.986–0.998

* Represent interaction.
